# Quality of home-based rapid HIV testing by community lay counsellors in a rural district of South Africa

**DOI:** 10.7448/IAS.16.1.18744

**Published:** 2013-11-14

**Authors:** Debra Jackson, Reshma Naik, Hanani Tabana, Mogiluxmi Pillay, Savathee Madurai, Wanga Zembe, Tanya Doherty

**Affiliations:** 1 School of Public Health, University of the Western Cape, Cape Town, South Africa; 2 Health Systems Research Unit, South African Medical Research Council, Cape Town, South Africa; 3 Global Clinical and Viral Laboratories, Durban, South Africa

**Keywords:** home-based HIV testing and counselling, sensitivity, specificity, rural, South Africa, quality assurance, rapid HIV tests

## Abstract

**Introduction:**

Lack of universal, annual testing for human immunodeficiency virus (HIV) in health facilities suggests that expansion of HIV testing and counselling (HTC) to non-clinical settings is critical to the achievement of national goals for prevention, care and treatment. Consideration should be given to the ability of lay counsellors to perform home-based HTC in community settings.

**Methods:**

We implemented a community cluster randomized controlled trial of home-based HTC in Sisonke District, South Africa. Trained lay counsellors conducted door-to-door HIV testing using the same rapid tests used by the local health department at the time of the study (SD Bioline and Sensa). To monitor testing quality and counsellor skill, additional dry blood spots were taken and sent for laboratory-based enzyme-linked immunosorbent assay (ELISA) testing. Sensitivity and specificity were calculated using the laboratory result as the gold standard.

**Results and discussion:**

From 3986 samples, the counsellor and laboratory results matched in all but 23 cases. In 18 cases, the counsellor judged the result as indeterminate, whereas the laboratory judged 10 positive, eight negative and three indeterminate, indicating that the counsellor may have erred on the side of caution. Sensitivity was 98.0% (95% CI: 96.3–98.9%), and specificity 99.6% (95% CI: 99.4–99.7%), for the lay counsellor field-based rapid tests. Both measures are high, and the lower confidence bound for specificity meets the international standard for assessing HIV rapid tests.

**Conclusions:**

These findings indicate that adequately trained lay counsellors are capable of safely conducting high-quality rapid HIV tests and interpreting the results as per the kit guidelines. These findings are important given the likely expansion of community and home-based testing models and the shortage of clinically trained professional staff.

## Introduction

Knowledge of human immunodeficiency virus (HIV) serostatus has been advocated as a prerequisite for access to care and support and increasingly as a prevention measure in its own right [1]. HIV testing and counselling (HTC) comprise the most widely accepted approach for promoting knowledge of serostatus. Despite efforts to increase availability and access to HTC in health facilities in South Africa, the Human Sciences Research Council (HSRC)/Nelson Mandela third national household survey in 2008 found that only 50.8% of participants aged 15 years or older had ever had an HIV test, and 24.7% of those aged 15–49 had a test in the last 12 months and knew their results [2].

Many barriers to HIV testing have been described, including long waiting times, lack of transport to reach facilities, concerns about staff confidentiality and drawbacks to testing due to health systems factors, such as insufficient stock of HIV test kits [3]. Recently, in a number of countries in sub-Saharan Africa, community-based approaches to HTC have been implemented to increase HTC uptake, with positive results [4–8]. However, only one of these studies was a randomized trial, leading the Cochrane Review to conclude that there is insufficient evidence to recommend large-scale implementation of this approach [5]. This highlights the urgent need for rigorous research to guide policy on HTC approaches for settings with high HIV prevalence.

An expansion of community and home-based testing will likely require an increased use of lay counsellors due to shortages of trained professional staff. In addition, the World Health Organization (WHO) recommends that the quality of rapid testing should be included in the evaluation of any HTC programme [9]. This would include evaluations of home-based HIV testing by lay counsellors. One study on the quality of home-based rapid testing on venous whole-blood samples taken by paramedical staff in Malawi showed sensitivity of 99.6% and specificity of 100.0% [10]. This article describes the sensitivity and specificity of rapid HIV testing using finger prick blood, performed by lay counsellors as part of a randomized controlled trial in a rural district of South Africa.

## Methods

This analysis was part of a community cluster randomized controlled trial to evaluate home-based HTC conducted by the South African Medical Research Council and the University of the Western Cape, in collaboration with the Sisonke District Department of Health (the Good Start study, trial no. ISRCTN31271935).

The trial was conducted in 16 communities in the Umzimkhulu subdistrict of the Sisonke district, KwaZulu-Natal, between September 2009 and January 2011. Umzimkhulu is one of the poorest rural areas in South Africa, where 77% of households live below the poverty line [11]. Maps showing population numbers based on the Statistics South Africa (StatsSA) census were used to demarcate clusters with approximately 150 households each. A baseline survey of 18+ year olds (age range 18–100) prior to the start of the trial showed “ever” HIV testing rates in the households to be 32% [12].

Prior to the study, counsellors completed a 10-day nationally accredited course in HTC, during which they learned how to conduct both of the rapid HIV tests used in the district protocol. They spent a further three months shadowing facility lay counsellors and gaining nurse-supervised testing experience at local health facilities. Additionally, they received a one-day training on obtaining and packaging dried blood spot (DBS) samples from laboratory technicians. After an initial community mobilization, lay counsellors in the intervention clusters proceeded from house to house in a systematic manner until they covered every household in their cluster. At each house, they offered HTC to any household member who met the eligibility criteria and was willing to be tested. Inclusion criteria for the intervention included living in a household in an intervention cluster, being 18 years of age (or 14–17 years of age with parental or guardian permission) and providing written informed consent for the HTC.

Counsellors used the same rapid HIV screening tests (SD Bioline (Standard Diagnostics Inc., Kyonggi-do, South Korea) with confirmatory SENSA (Sensa Tri-line HIV 1/2/0; Hitech Healthcare Ltd, Beijing, China)) that were used by district health facilities at the time of the study. Rapid tests were done on blood obtained from finger pricks. For the purpose of monitoring test quality and counsellor skill, additional DBSs from the same finger prick were taken for laboratory-based enzyme-linked immunosorbent assay (ELISA) testing. The DBSs were dried, packaged and then transported to Global Clinical and Viral Laboratory in Durban, KwaZulu-Natal, on a weekly basis. A sample of HIV-negative results (62.5%) and all HIV-positive, indeterminate and discordant-couple results from the field were sent for laboratory-based ELISA testing. The laboratory used the following testing algorithm: all samples were screened using Vironostika HIV Uni-Form II Plus O (Biomerieux, Marcy L'Etoile, France), quality control DBS blots obtained from the US Centers for Disease Control and Prevention were run with each batch of test samples, all samples that tested positive were confirmed by testing on SD Bioline ELISA (Pantech, Durban, South Africa) and tie breakers (where the SD Bioline did not correlate with the initial screening assay, Vironostika HIV Uni-Form II) were run on a Roche Elecsys System (Roche Diagnostics India, Mumbai, India) for final verification. Two of three results were taken as the final interpretation, in accordance with WHO guidelines.

Along with basic demographic data, and a unique participant and laboratory identification (ID) number, counsellor test results were entered into a cell phone used for data collection [13] (www.mobenzi.com) immediately following testing. These data were then downloaded from a central server to an Excel file. The laboratory database was also received as an Excel file. We merged the two Excel files based on the laboratory ID number and identified a total of 3986 samples with both a counsellor and laboratory-based test result. Included in the 3986 were some repeat tests from the same individual, since repeat testing was offered to HIV-negative individuals 3–6 months following the initial test. We then used V-lookup and field-matching formulas in Excel to identify discrepancies in test results. A 2×2 table was constructed comparing counsellor results to laboratory results. Sensitivity and specificity were calculated using the laboratory test as the gold standard [14], and 95% confidence intervals were calculated using the Wilson method [15].

All participants gave oral informed consent for study participation and written informed consent for the actual HIV testing, in accordance with local district procedures. Information sheets were given to prospective participants in the local languages (Zulu or Xhosa) with explanations about the home-based counselling and testing intervention. Ethics approval was obtained from the Ethics Committee of the South African Medical Research Council (EC09-003).

## Results and discussion

During the period of the study, counsellors tested 5086 unique individuals, with a rate of living with HIV of 9.5% [16]. Of the 3986 matched samples, the counsellor results were in concordance with the laboratory results in the vast majority of cases (99.4%). There were only 23 cases where results did not match. Of these, 10 were cases where the counsellor had an indeterminate result and the laboratory found the sample to be positive. These are interpreted as cases where the counsellor was erring on the side of caution (e.g. waiting for confirmatory laboratory results before giving a positive result to the client). In cases where field rapid tests were conflicting, the counsellor would advise the client that blood would be referred to the lab for confirmation of status and results returned to the client within 2–4 weeks. There were eight cases where the counsellor had an indeterminate result and the laboratory found the DBS sample to be negative. In three cases, the laboratory had an indeterminate result when the counsellor result was positive (*n*=2) or negative (*n*=1); however, we were unable to reach the clients for retesting and verification. Finally, there were two cases where the counsellor had a positive result and the laboratory had a negative result. These are the only two cases that would be considered critical errors. Both clients were contacted and told about their laboratory-confirmed results.

Table 1 compares the results of the lay counsellor field-based rapid tests and the laboratory-based ELISA results. The indeterminate and negative results are combined in this table to allow calculation of sensitivity and specificity.

**Table 1:** Comparison of lay counsellor field-based rapid test results with laboratory-based enzyme-linked immunosorbent assay (ELISA) results

	ELISA laboratory result		
		
Lay counsellor result	Living with HIV	HIV-negative or indeterminate	Total
Living with HIV	481	4	485
HIV-negative or indeterminate	10	3491	3501
Total	491	3505	3986

The results suggest a sensitivity of 98.0% (95% CI: 96.3–98.9%) and a specificity of 99.6% (95% CI: 99.4–99.7%) for the lay counsellor field-based rapid tests. Both measures are high, and the lower confidence bound for specificity meets the international standard for assessing HIV rapid tests of above 98% (US Food and Drug Administration) [17]. Also of note, only two needle sticks or other injuries occurred during the intervention.

## Discussion

Our study of lay counsellors doing home-based rapid HIV testing in a rural community found a sensitivity of 98.0% and specificity of 99.6%. Compared to the home-based HTC study reported by Molesworth [10] from Malawi, which used venous whole blood and trained paramedical professionals, this study used home-based lay counsellors using finger prick blood in a more operational rural field setting. A study by Molesworth *et al*. [10] found sensitivity of 99.6% and sensitivity of 100%, which is similar to and within the confidence limits of our results.

In our analysis, we included inconclusive results with the negative results in order to calculate sensitivity and specificity, which has been noted to decrease specificity [18]. Inconclusive results (e.g. conflicting rapid test results) in the field suggest the need for routine laboratory backup for home-based programs to address both routine quality assurance as well as inconclusive field-based results.

Interestingly, other studies in South Africa have highlighted quality issues with HIV rapid tests performed by clinic-based nurses. These studies have reported sensitivity and specificity in the ranges of 92.5–97.3% and 97.6–98.2%, respectively [19, 20]. Our home-based lay counsellors achieved better results than these clinic-based studies with professional nurses. This may be due to the fact that our lay counsellors had extensive training and practical clinic-based experience prior to moving to the field, which enhanced performance and adherence to testing protocols, even in a difficult field-based setting. Importantly, the limited number of needle sticks that occurred in a non-facility field environment also speaks to the ability of lay counsellors to maintain personnel safety within a home-based setting.

The government of South Africa is currently embarking on revitalization of the primary healthcare system [21], which includes the creation of community outreach teams, including a specially trained cadre of community health workers who provide a package of health services within households. Although a national policy allowing finger prick testing to be performed by lay health workers was passed in 2010 [22], HIV testing is currently not in the scope of practice of these community health workers. Given the low rates of facility-based HIV testing and the success of home-based HIV testing in achieving high rates of coverage [16], the high quality of testing demonstrated by this study provides a strong impetus for HIV testing to be considered as a role of community health workers.

## Conclusions

These findings indicate that adequately trained lay counsellors are capable of safely conducting high-quality rapid HIV tests and interpreting the results as per the kit guidelines. These findings are important given the likely expansion of community and home-based testing models and the shortage of clinically trained professional staff. This evidence supports a recent change to South African government regulations stating that trained lay counsellors can conduct finger pricks to obtain small quantities of blood for testing. This will have important implications for the expansion of HTC services to community and home-based settings as a potential component of the South African programme to revitalize primary healthcare. This PHC revitalization programme includes increasing the deployment of community health workers. However, to date home-based HTC has not been included in the package of services provided by community health workers. This study suggests that home-based HTC by community health workers could be used to assist in expanding home-based HIV testing to reach rural communities throughout South Africa, as well as similar communities across Africa.
